# Facility delivery and postnatal care services use among mothers who attended four or more antenatal care visits in Ethiopia: further analysis of the 2016 demographic and health survey

**DOI:** 10.1186/s12884-019-2216-8

**Published:** 2019-02-11

**Authors:** Gedefaw Abeje Fekadu, Fentie Ambaw, Seblewongiel Ayenalem Kidanie

**Affiliations:** 1 School of Public Health, College of Medicine and Health Sciences, Bahir Dar University, Bahir Dar, Ethiopia; 2 African Mental Health Research Initiative (AMARI) post-doctoral fellow, Addis Ababa University, Addis Ababa, Ethiopia; 3 Department of Social Work, Faculty of Social Sciences, Bahir Dar University, Bahir Dar, Ethiopia

**Keywords:** Antenatal care, Facility delivery, Postnatal care, Quality of care, Ethiopia

## Abstract

**Background:**

Antenatal care provides the best opportunity to promote maternal and child health services use. But many Ethiopian mothers deliver at home and fail to attend postnatal care. Therefore, this study was done to identify factors associated with health facility delivery among mothers who attended four or more antenatal care visits. The study was also intended to identify factors associated with postnatal care service use among mothers who delivered at home after four or more antenatal care visits.

**Methods:**

This study used the 2016 Ethiopian Demographic and Health Survey data. Two thousand four hundred fifteen women who attended four or more antenatal care visits were included to identify factors associated with health facility delivery after four or more antenatal care visits. Among them, 1055 mothers delivered at home. These women were included to identify factors associated with postnatal care service use. Stata 15.1 was used to analyze the data. Multivariable logistic regression model was fitted to identify associations between the outcome and predictor variables.

**Results:**

Among women who had four or more antenatal care visits, 56% delivered at health facility. Mothers with secondary or higher level of education (AOR = 2.9; 95% CI = 1.6–5.3), urban residents (AOR = 3.4; 95% CI = 1.9–6.1), women with highest wealth quintile (AOR = 2.7; 95% CI = 1.5–4.8), and working women (AOR = 1.6; 95% CI = 1.2–2.3) had higher odds of delivering at health facilities. High birth order (AOR = 0.5; 95% CI = 0.3–0.7) was negatively associated with a lower likelihood of health facility delivery. Among women who delivered at home, only 8% received postnatal care within 42 days after delivery. Only the content of care received during antenatal care visits (AOR = 1.40; 95% CI = 1.1–1.8) was significantly associated with postnatal care attendance.

**Conclusion:**

Women with lower socio-economic status had lower odds of giving birth at health facility even after attending antenatal care. The more antenatal care components a mother received, the higher her probability of delivering at health facility. Similarly, postnatal care attendance was higher among women who had received more antenatal care components.

## Background

Although preventable with proven, cost-effective interventions, maternal mortality is global public health problem [1, 2]. Antenatal care (ANC), skilled attendant at delivery and postnatal care (PNC) are some of these effective interventions [3–9].

Antenatal care is an opportunity for health care providers to detect and treat causes of maternal mortality. When the number of ANC visits increases, the woman is expected to acquire more knowledge and develop better attitude about using other maternal health services [10–13]. Studies recommended four or more focused visits for antenatal care to improve maternal and neonatal outcomes [14, 15]. A systematic review and meta-analysis of evidences in sub-Saharan African countries documented that mothers who attended at least four ANC visits were seven times more likely to deliver in health facility [16]. Another study among four African countries reported a positive effect of the number of ANC consultations with use of a skilled birth attendant [17]. Studies in Colombia, Nepal, Bangladesh, Zambia, and Kenya showed similar finding [18–22]. A study conducted in Debremarkos town, Northwest Ethiopia, reported that women who attended four or more ANC visits were five times more likely to deliver in health facility compared with women who attended fewer visits [23].

Ethiopia had developed many strategies and programs to improve maternal health services use. For example, family planning, ANC, facility delivery, and PNC services are provided free of charge [10, 24–27]. Regardless of these efforts, health facility delivery service use (26%) and PNC uptake (17%) remained very low. This low facility delivery and PNC services use was observed although there was improvement in ANC service use from 27% in 2000 to 62% in 2016) [28]. Improvement in ANC service use is expected to improve facility delivery and postnatal care services use by enhancing behaviors beneficial to the mother and her child including delivering in health facility and using postnatal care services. Why institutional delivery and postnatal care services use remained low while antenatal care services use is relatively high in Ethiopia was not clear. The available studies in Ethiopia are small to answer this question. Therefore, this study was done to identify factors associated with health facility delivery service use among mothers who attended four or more ANC visits. The study also intended to identify factors associated with PNC service use among mothers who delivered at home after attending four or more ANC visits. Answering this question will help policymakers and programmers to improve the continuity of care from antenatal to facility delivery and postnatal care service use.

## Methods

### Data

This study used the 2016 Ethiopian Demographic and Health Survey data, which was collected by the Central Statistical Agency (CSA), Ethiopia and the DHS Program, ICF [42]. The 2016 EDHS sample design had two stages. In the first stage, each region was stratified into urban and rural areas. Then, clusters were selected from both rural and urban areas using a probability proportional to size allocation. A list of all the households was prepared in all the selected clusters. In the second stage, a fixed number of households were selected systematically from the selected clusters. Then, all women age 15–49 years in the selected households were included.

The following criteria were used to select women to be considered in the analysis:

- Women who had at least one birth five years before the interview
- Women who attended four or more antenatal care visits during the most recent pregnancy

**Fig. 1:**
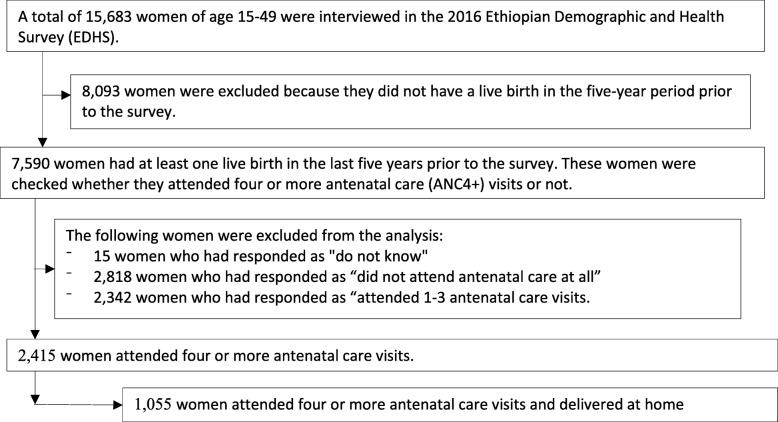
Schematic presentation of sample selection

Of the total 15,683 women included in the 2016 EDHS, 2415 attended four or more ANC visits for the most recent birth. These women were included in the analysis to identify factors associated with facility delivery. From these, 1055 mothers delivered at home. Only these women were included to identify factors associated with postnatal care service use among mothers who delivered at home after four or more ANC visits (Fig. 1). Women who delivered at home were the interest of this analysis for PNC service use because women who delivered at health facility were more likely to receive postnatal care even though the care was likely to be initiated by health care providers and thus does not measure women’s health seeking behavior [29–32].

### Measurements

The outcome variables for this analysis were place of delivery and postnatal care attendance.

The independent variables for this study were:

- Socio-demographic characteristics: maternal age at the time of birth of the most recent child, religion, educational status, marital status, place of residence (urban-rural), household wealth quintile and working status of the mother.
- Fertility related characteristics: birth order of the recent child, and whether the pregnancy was wanted or not.
- ANC service-related characteristics: mother’s perceived distance of the nearest health facility, components of antenatal care- measured by computing a summative composite score from the following antenatal components: blood pressure measurement taken, urine sample taken, blood sample taken, mother told about pregnancy complications, mother told about birth preparedness plan, and mother received nutrition counseling. For each of the component indicators, a woman received a point if she received the component. A total composite score was created by summing up the points for all six indicators. The total score ranged from zero to six where zero represents that the woman received none of the six antenatal care components and six when the woman received all components.

### Analytical methods

Data were analyzed using Stata 15.1. Sample weight was applied when computing frequencies. Descriptive statistics was used to describe the characteristics of mothers. Multicollinearity was checked using variance inflation factors (VIF). Binary logistic regression was used to identify predictors of health facility delivery and postnatal care service use among mothers who attended four or more ANC visits. All variables entered in the bivariate model were entered to the multivariable model.

## Results

### Sample characteristics

A total of 2415 mothers who had attended four or more antenatal care visits for the most recent birth in the five years before the survey were included in the analysis. The mean age of the mothers was 26.9 years. About two-third of the mothers were rural residents. Almost all (93%) were either married or in union. More than half (69%) were Christians in terms of religion. Regarding the level of education, about half of the mothers (48%) did not attend formal education. The majority of mothers (66%) had no formal job. Considering the wealth index, 30% mothers were in the richest wealth quintile. Other characteristics of the women are shown in table below (Table 1).

**Table 1:** Socio-demographic characteristics of mothers who attended four or more ANC visits for the most recent birth within five years before the survey, Ethiopia, 2016

Characteristics	Percent
Age of the mother at birth in years	
Mean (SD)	26.9 (6.2)
Educational level	
No education	47.9
Primary	34.3
Secondary& above	17.8
Religion	
Christian	69.1
Other	30.9
Marital status	
Married/in union	92.8
Other	7.2
Place of residence	
Urban	25.2
Rural	74.8
Wealth index	
Poorest	12.6
Poorer	17.3
Middle	18.5
Richer	21.4
Richest	30.2
Mother’s working status	
No	65.8
Yes	34.2
Birth order	
First	24.9
Second to fourth	45.0
Five or more	30.1
Perceived distance to health facility	
Big problem	45.9
Not a big problem	54.1
Recent birth wanted	
Wanted then	76.7
Wanted later	16.3
Wanted no more	7.0

### Health facility delivery among women who attended four or more antenatal care visits

Among women who attended four or more ANC visits, 56% delivered at health facility. The table below shows the characteristics of women who delivered at health facility after four or more ANC visits (Table 2).

**Table 2:** Characteristics of women who delivered at health facilities after four or more ANC visits for the most recent birth in the five years before the survey, Ethiopia, 2016 DHS

Characteristics of mothers	Percent
Age of mother (year)	26.7 (6.0)
Mean (SD)	
Educational level	
No education	42.6
Primary	57.3
Secondary & above	91.3
Religion	
Christian	59.4
Other	49.3
Marital status	
Married/in union	56.2
Other	57.4
Place of residence	
Urban	91.9
Rural	44.3
Wealth index	
Poorest	34.3
Poorer	40.7
Middle	43.2
Richer	49.3
Richest	87.5
Mother’s working status	
No	49.4
Yes	69.5
Perceived distance to health facility	
Big problem	43.8
Not a big problem	66.9
Birth order	
First	73.9
Second to fourth	58.1
Fifth or more	39.0
Wantedness of the child	
Wanted then	55.9
Wanted later	57.5
Wanted no more	57.6

### Factors associated with health facility delivery after four or more antenatal care visits

The binary logistic regression model showed that educational status, place of residence, wealth index, mother’s working status, birth order, and components of ANC were significantly associated (*p* < 0.05) with health facility delivery among women who attended four or more ANC visits. Mothers who had completed secondary and tertiary level of education had 2.9 times (95% CI: 1.6–5.3) higher odds of health facility delivery compared to mothers who had no formal education. Urban resident mothers had 3.4 times (95% CI: 1.9–6.1) higher odds of health facility delivery compared to rural women. Women in the richest wealth quintile had 2.7 times (95% CI: 1.5–4.8) higher odds of using health facility delivery compared to mothers in the poorest quintile.

Mothers who were working outside home at the time of interview had 1.6 times (95% CI: 1.2–2.3) higher odds of delivering at health facility compared to non-working mothers. Women with 2–4 birth orders had 50% (95% CI: 0.3–0.7%) lower odds of delivering at health facility compared to first-order births. Similarly, women with birth order of five or more had 70% (95% CI: 0.2–0.6) lower odds of delive ring at health facility compared to women with first-order births. An increase in receiving components of ANC improves the odds of health facility delivery by 30% (95% CI: 1.2–1.4) (Table 3).

**Table 3:** Factors associated with health facility delivery among women who attended four or more ANC visits, Ethiopia, 2016

Characteristics of Mothers	Facility Delivery	
	COR(95% CI)	AOR(95% CI)
Age of the mother at birth	1.0 (0.9–1.0)	1.0 (1.0–1.1)
Educational level		
No education	1.0	1.0
Primary	1.8 (1.4–2.3)***	1.1 (0.8–1.5)
Secondary& above	14.1 (8.8–22.7)***	2.9 (1.6–5.3)***
Religion		
Christian	1.5 (1.1–2.1)***	1.00
Other	1.0	1.1 (0.8–1.6)
Marital status		
Married/in union	1.0	1.3 (0.7–2.2)
Other	1.0 (0.6–1.7)	1.0
Place of residence		
Urban	14.2 (9.5–21.3)***	3.4 (1.9–6.1)***
Rural	1.0	1.0
Wealth index		
Poorest	1.00	1.0
Poorer	1.3 (0.8–2.1)	1.3 (0.8–2.2)
Middle	1.5 (0.9–2.3)	1.3 (0.8–2.1)
Richer	1.9 (1.2–2.9)***	1.4 (0..9–2.2)
Richest	13.4 (8.1–22.2)***	2.7 (1.5–4.8)***
Mother’s working status		
No	1.0	1.0
Yes	2.3 (0.8–1.2)	1.6 (1.2–2.3)***
Birth order		
First	4.4 (3.1–6.3)***	0.5 (0.3–0.7)***
2–4	2.2 (1.6–2.9)***	0.3 (0.2–0.6)***
Five or more	1.0	1.0
Perceived distance to health facility		
Big problem	1.0	1.0
Not a big problem	2.6 (1.9–3.5)***	1.3 (1.0–1.7)
Wanted status of child		
Wanted then	1.00	1.0
Wanted later	1.1 (0.8–1.4)	1.0 (0.7–1.4)
Wanted no more	1.1 (0.7–1.7)	1.5 (0.9–2.5)
Receiving components of ANC	1.5 (1.4–1.6)***	1.33 (1.2–1.4)***

*** refers to significant at *p* less than or equal to 0.05

### Characteristics of women who delivered at home after four or more antenatal care visits

Among mothers who attended four or more ANC visits, 44% delivered at home. Table 4 below shows the characteristics of women who delivered at home after four or more ANC visits.

**Table 4:** Socio-demographic characteristics of mothers who delivered the most recent child at home after four or more antenatal care visits, Ethiopia, 2016

Characteristics of Mothers	Home Delivery
	Percent
Age of the mother in years	27.2 (6.0)
Mean (*SD*)	
Educational level	
No education	62.9
Primary	33.5
Secondary& above	3.6
Religion	
Christian	64.2
Other	35.8
Marital status	
Married/in union	93.0
Other	7.0
Place of residence	
Urban	4.7
Rural	95.3
Wealth index	
Poorest	19.0
Poorer	23.5
Middle	24.1
Richer	24.8
Richest	8.6
Mother’s working status	
No	76.1
Yes	23.9
Perceived distance to health facility	
Big problem	59.0
Not a big problem	41.0
Number of ANC components received Mean(±SD)	3.8(±1.7)

### Postnatal care (PNC) among mothers who delivered at home after four or more ANC visits

Among women who gave birth at home after attending four or more ANC visits, only 8% (95% CI: 6.1–11.5%) attended postnatal care. There was variation in PNC attendance by socio-demographic characteristics (Table 5).

**Table 5:** Postnatal care attendance for the most recent birth among women who delivered at home after four or more antenatal care visits by socio-demographic characteristics, Ethiopia, 2016

Characteristics of Mothers	Percent PNC Attended
Age of the mother in years	
Mean (*SD*)	28.3 (6.2)
Educational level	
No education	8.5
Primary	7.9
Secondary & above	11.8
Religion	
Christian	9.9
Other	5.7
Marital status	
Married/in union	8.3
Other	9.3
Place of residence	
Urban	24.0
Rural	7.6
Wealth index	
Poorest	7.3
Poorer	8.6
Middle	11.1
Richer	7.5
Richest	20.5
Mother’s working status	
No	7.5
Yes	11.3
Perceived distance to health facility	
Big problem	5.2
Not a big problem	13.1
Number of ANC components received Mean(±SD)	5(±1.3

### Factors associated with postnatal care attendance among women who delivered at home after four or more antenatal care visits

From all the variables included in the multivariable logistic regression model, only the number of ANC components a woman received during antenatal care was significantly associated with postnatal care services use. A one unit increase of one component of ANC improves the odds of using postnatal care service by 40% (95% CI: 10–80%) (Table 6).

**Table 6:** Factors associated postnatal care attendance among mothers who delivered at home after four or more ANC visits, Ethiopia, 2016

Characteristics of Mothers	Postnatal	
	Care Attendance	
	COR(95% CI)	AOR(95% CI)
Age of the mother at birth	1.0 (1.0–1.1)	1.0 (1.0–1.1)
Educational level		
No education	1.00	1.0
Primary	0.9 (0.5–1.8)	0.9 (0.4–1.9)
Secondary& above	1.4 (0.4–5.3)	0.7 (0.2–2.9)
Religion		
Christian	1.8 (0.8–4.1)	0.8 (0.4–1.7)
Other	1.0	1.0
Marital status		
Married/in union	1.0	1.0
Other	1.1 (0.4–3.0)	0.1.0 (0.4–2.6)
Place of residence		
Urban	3.8 (1.5–9.9)***	1.3 (0.5–3.8)
Rural	1.0	1.0
Wealth index		
Poorest	1.0	1.0
Poorer	0.4 (0.1–1.4)	0.4 (0.1–1.4)
Middle	1.6 (0.6–3.8)	1.4 (0.5–3.5)
Richer	1.0 (0.4–2.7)	0.8 (0.3–2.2)
Richest	3.2 (1.3–7.9)***	1.8 (0.7–4.8)
Mother’s current working Status		
No	1.0	1.0
Yes	1.6 (0.9–2.8)	1.4 (0.8–2.7)
Birth order		
First	0.6 (0.2–1.7)	1.5 (0.4–5.0)
2–4	1.2 (0.6–2.4)	1.0 (0.2–4.7)
Five or more	1.0	1.0
Perceived distance to health facility		
Big problem	1.0	1.0
Not a big problem	2.7 (1.5–5.0)***	1.9 (1.0–3.8)
Wanted status of child		
Wanted then	1.0	1.0
Wanted later	1.5 (0.7–3.3)	1.30.6–3.1)
Wanted no more	1.8 (0.7–4.8)	1.6 (0.5–4.5)
Receiving components of ANC	1.5 (1.2–1.9)***	1.40 (1.1–1.8)*

***p < 0.05

## Discussion

### Health facility delivery among women who attended four or more antenatal care visits

The analysis revealed that among women who attended four or more ANC visits, over half of them gave birth at health facility. This level of health facility delivery service use is low compared to the national target of 95% deli very attendance with a skilled provider by 2020 [33, 34]. Currently available evidences reported that mothers who attended four or more ANC visits are more likely to be informed and aware of the importance of health facility delivery. These women already won the challenges of visiting health facility. Therefore, they are expected to give birth in health facilities. However, the finding of this study contradicts this assumption. The possible reasons for low health facility delivery service use among women who attended four or more ANC visits may be the poor quality of information provided during antenatal care sessions. The other reasons may be the women’s perceived poor quality of delivery care provided at the health facilities. If women rated the quality of delivery service low, they are less likely to give birth at health facility although they attended ANC.

Mothers who completed secondary or higher level of education had higher odds of giving birth at health facility compared to mothers who did not attend formal education. This finding was similar with studies conducted in Nepal, Cambodia, and a meta-analysis in Ethiopia [19, 21, 35]. Higher level of education is associated with better information processing skill, improved cognitive skills and values, exerting effects on health seeking behavior through different pathways. These pathways include higher level of awareness and better knowledge of maternal health services and enhanced level of autonomy. Education increases feelings of self-worth and confidence which all improve health service use [36, 37].

Urban mothers had higher odds of giving birth at health facility compared to rural mothers. This finding was similar with studies conducted in Ethiopia and Ghana [35, 38]. Urban women have better exposure to media regarding the benefits of facility delivery. Cultural barriers related with health service use are less prevalent in urban areas compared to rural areas [39]. Similarity, accessibility and quality of health services may be better in urban settings. Urban women are also more educated than rural woman [40–42].

The analysis revealed that women in the richest wealth quintile had higher odds of giving birth at health facilities compared to poorest women. This finding was consistent with other studies [19, 21, 43]. The cost of health facility delivery may not be the reason in Ethiopia because all maternal health services are provided free of charge. However, other costs such as transport, time spent by the mother and family members at the hospital, and fear of unofficial payments, could help explain the lower odds of giving birth at health facilities among the poor. Working mothers had higher odds of delivering at health facility. This finding was consistent with previous reports [44]. The reason for this is that working mothers are more likely to be autonomous for health services use. They are also more likely to receive information at work that promotes health facility delivery service use [45–47].

Second and higher order births had lower odds of being delivered at health facility compared to first order births. This finding was consistent with other studies [19, 21] but contradicts a study conducted in Ethiopia. Higher order births are more likely to be unwanted, which in turn could affect the use of maternal health services [48–51]. Another possible explanation for lower odds of being delivered at health facilities for higher order births is that mothers could be fearful during first birth and hence more likely to use health facility for delivery for the first birth than subsequent births [52, 53]. In addition, women in the younger generation initiating childbearing are more educated than the older generation.

Mothers who received more ANC components had higher odds of delivering at health facility. This finding was similar with studies conducted in Mexico and Cambodia [21, 54]. Currently available evidences indicate that better quality ANC service is associated with higher health facility delivery service use. The number of ANC components a woman received during ANC can be considered as a proxy indicator for the quality of ANC.

### Factors associated with postnatal care attendance among women who delivered at home after four or more ANC visits

Postnatal care attendance among mothers who delivered at home after four or more ANC visits was very low compared with the national child survival strategy and the health sector transformation plan targets for 2020 [33, 34]. Among women who attended four or more ANC visits and delivered at home, only 8% attended postnatal care. This implies that mothers were not using postnatal care services although they managed all the challenges of attending the recommended number of ANC visits.

Mothers who received more ANC components had higher odds of attending PNC. Receiving more ANC components implies that the mother is more likely to be informed about complications that may occur after delivery and thus recognize the importance of timely postnatal care.

## Conclusions

Health facility delivery service use among women who attended four or more ANC visits was still low in Ethiopia. Women with higher socioeconomic status (richest, working, educated, and urban) had higher odds of delivering at health facility. In addition, the more ANC components women received, the more likely she give birth at health facility and attend postnatal care services. Further studies on how continuity of care following ANC is associated with socio-economic status and components of ANC care are required.

## Abbreviations

ANC: Antenatal care
AOR: Adjusted odds ratio
CI: Confidence interval
CSA: Central statistical agency
EDHS: Ethiopian demographic and health survey
HIV: Human immuno-deficiency virus
PNC: Postnatal care
VIF: Variance inflation factors
